# Cerebrospinal fluid metabolic profiling reveals divergent modulation of pentose phosphate pathway by midazolam, propofol and dexmedetomidine in patients with subarachnoid hemorrhage: a cohort study

**DOI:** 10.1186/s12871-022-01574-z

**Published:** 2022-01-27

**Authors:** Yi-Chen Li, Rong Wang, Ji-Ye A, Run-Bin Sun, Shi-Jie Na, Tao Liu, Xuan-Sheng Ding, Wei-Hong Ge

**Affiliations:** 1 Department of Pharmacy, Nanjing Drum Tower Hospital Affiliated to Nanjing University Medical School, Nanjing, 210008 China; 2 Department of Neurosurgery, Nanjing Drum Tower Hospital Affiliated to Nanjing University Medical School, Nanjing, 210008 China; 3 Nanjing Medical Center of Clinical Pharmacy, Nanjing, 210008 China; 4 Key Laboratory of Drug Metabolism and Pharmacokinetics, China Pharmaceutical University, Nanjing, 210009 China; 5 Department of Basic Medicine and Clinical Pharmacy, China Pharmaceutical University, Nanjing, 210009 China

**Keywords:** Subarachnoid hemorrhage, Sedation, Midazolam, Propofol, Dexmedetomidine, Metabolomics, Outcome

## Abstract

**Background:**

Agitation is common in subarachnoid hemorrhage (SAH), and sedation with midazolam, propofol and dexmedetomidine is essential in agitation management. Previous research shows the tendency of dexmedetomidine and propofol in improving long-term outcome of SAH patients, whereas midazolam might be detrimental. Brain metabolism derangement after SAH might be interfered by sedatives. However, how sedatives work and whether the drugs interfere with patient outcome by altering cerebral metabolism is unclear, and the comprehensive view of how sedatives regulate brain metabolism remains to be elucidated.

**Methods:**

For cerebrospinal fluid (CSF) and extracellular space of the brain exchange instantly, we performed a cohort study, applying CSF of SAH patients utilizing different sedatives or no sedation to metabolomics. Baseline CSF metabolome was corrected by selecting patients of the same SAH and agitation severity. CSF components were analyzed to identify the most affected metabolic pathways and sensitive biomarkers of each sedative. Markers might represent the outcome of the patients were also investigated.

**Results:**

Pentose phosphate pathway was the most significantly interfered (upregulated) pathway in midazolam (*p* = 0.0000107, impact = 0.35348) and propofol (*p* = 0.00000000000746, impact = 0.41604) groups. On the contrary, dexmedetomidine decreased levels of sedoheptulose 7-phosphate (*p* = 0.002) and NADP (*p* = 0.024), and NADP is the key metabolite and regulator in pentose phosphate pathway. Midazolam additionally augmented purine synthesis (*p* = 0.00175, impact = 0.13481) and propofol enhanced pyrimidine synthesis (*p* = 0.000203, impact = 0.20046), whereas dexmedetomidine weakened pyrimidine synthesis (*p* = 0.000000000594, impact = 0.24922). Reduced guanosine diphosphate (AUC of ROC 0.857, 95%CI 0.617–1, *p* = 0.00506) was the significant CSF biomarker for midazolam, and uridine diphosphate glucose (AUC of ROC 0.877, 95%CI 0.631–1, *p* = 0.00980) for propofol, and succinyl-CoA (AUC of ROC 0.923, 95%CI 0.785–1, *p* = 0.000810) plus adenosine triphosphate (AUC of ROC 0.908, 95%CI 0.6921, *p* = 0.00315) for dexmedetomidine. Down-regulated CSF succinyl-CoA was also associated with favorable outcome (AUC of ROC 0.708, 95% CI: 0.524–0.865, *p* = 0.029333).

**Conclusion:**

Pentose phosphate pathway was a crucial target for sedatives which alter brain metabolism. Midazolam and propofol enhanced the pentose phosphate pathway and nucleotide synthesis in poor-grade SAH patients, as presented in the CSF. The situation of dexmedetomidine was the opposite. The divergent modulation of cerebral metabolism might further explain sedative pharmacology and how sedatives affect the outcome of SAH patients.

## Background

Subarachnoid Hemorrhage (SAH) is a neurological emergency, and the mortality of poor-grade SAH patients is relatively high [1]. Agitation is one of the common complications of acute SAH [2–4], and is associated with worse long-term outcome in non-comatose SAH patients (Hunt–Hess I–IV) [5].

Sedation is the essential and major treatment of agitation in SAH [6, 7] and in neurocritical care units [8, 9]. Midazolam, propofol and dexmedetomidine are the most used sedatives [6, 8, 9]. Previous studies have demonstrated that sedatives might play roles in altering the outcome of the brain-injured patients; however, the influences of different agents are divergent [10]. Low dose dexmedetomidine, rather than no use, is significantly associated with higher rate of favorable outcome in poor-grade SAH patients [10, 11]. Dexmedetomidine also exerts brain protective effect in patients with intracranial aneurysm [12]. Levels of glial and neuronal injury markers are preserved in dexmedetomidine-treated patients while these markers significantly increased in patients treated with normal saline [12]. Meanwhile, most experimental and clinical studies also demonstrate the beneficial effect of propofol on secondary brain injury after SAH [10]. Propofol significantly reduces cerebral edema and improves neurological outcome of SAH rats [10]. And the cognitive function is better in propofol-treated SAH patients undergoing intracranial aneurysm clipping [10, 13]. Interestingly, on the contrary, another clinical study describes that the administration of propofol might be the detrimental [14]. Moreover, the administration of midazolam during acute phase is associated with unfavorable outcome after 6 months [14] and enhanced spreading depolarization [15] in SAH patients. Other studies, which mainly focus on dexmedetomidine and propofol, failed to link the utilization of sedatives with short- and long-term outcome in SAH patients [16–18].

It is still unclear whether and how agitation management affect the neurological outcome of the SAH patients [3]. Notably, metabolic derangement is found after the onset of experimental subarachnoid hemorrhage as well as in clinical settings [19–22], and is independent of cerebral ischemia [21]. Sedatives also interfere with brain metabolism; although whether and how the alteration of brain metabolism by sedatives is linked to the neuronal protection and patient prognosis is incompletely known.

Previous studies have proved that these three sedatives present metabolic suppression in generally anesthetized patients by decreasing cerebral blood flow and regulating cerebral metabolic rate [23–26]. Meanwhile, the cerebral metabolic alterations are directly associated with the consciousness of the patients. In brain-injured patients, positron emission computed tomography (PET) study shows a relationship between hypometabolism, neural network disconnections and deteriorated consciousness [27]. Midazolam-induced unconsciousness results from decreased widespread regional cerebral metabolic rates for glucose (rCMRglc); more importantly, the arousal, cognitive ability and motor activity gradually recover after the metabolism of each brain region is normalized [28]. Therefore, the detailed investigation of how sedatives alter brain metabolism in neuro-critically ill patients is essential in understanding drug action and applying the results into clinical practice.

Meanwhile, the three drugs display significant disparity in cerebral metabolic regulation, and even controversy exists. Clinical study has revealed that propofol and other sedatives differ in the alterations of plasma and cerebral metabolomic signature [29, 30]. In addition, the potency of dexmedetomidine in reducing whole brain metabolic rate is relatively higher than propofol in healthy subjects, suggesting their divergent regulation of brain metabolism [26]. Notably, the regulation of dexmedetomidine on cerebral blood flow and metabolic rate might be different in brain-injured patients, in which the former is reduced while the latter is meanwhile kept stable [25]. Another study shows no difference between midazolam and propofol in regulating cerebral glucose metabolism [31]. The insight into how these sedatives differentially modify the cerebral metabolic signature needs to be further investigated.

In clinical settings, although non-invasive PET and proton magnetic resonance (^1^HMR) spectroscopic approaches demonstrate how brain metabolism is changed in situ, they still have drawbacks. The identified compounds are limited, which is inadequate to provide sufficient metabolic information [27, 30]. Moreover, microdialysis, which is the feasible way to locally monitor how brain metabolizes, is not accessible to every patient, especially to those who do not undergo craniotomy [19, 32].

In the present study, we utilized cerebrospinal fluid (CSF) as the partial representative of cerebral metabolism, based on its exchange with extracellular space of the brain (ECSB), which is the interstitial fluid of the brain [33]. CSF could partly represent the fluctuation of metabolite concentrations in cytoplasm within the brain. CSF/ECSB exchange might provide a solution to observe the metabolic alterations in clinical cases, and CSF is more accessible. CSF could be the alternative to take a glimpse of how drug interact with brain metabolism and CSF composition. CSF/ECSB metabolic profile could be investigate through metabolomic way or focusing on specific compounds or pathways, and the former is more comprehensive. There has been a metabolomic research demonstrating that CSF composition is significantly divergent in anesthetic-induced coma from that of normal or pentylenetetrazol-treated epileptic rats [34]. Nevertheless, clinical studies of the gross CSF metabolome alterations in SAH patients after sedative treatment were absent [35]. Therefore, the present study was initiated to confirm the overall impact of sedatives to CSF metabolites in SAH patients.

In SAH patients, it would be difficult to distinguish where the CSF alterations come from— the sedatives or the disease itself— for SAH brings blood to subarachnoid space and involves CSF composition alterations. To solve this problem, we have demonstrated in our previous research that CSF composition patterns could be divided by clinical SAH severity (Hunt-Hess Scale), and is not associated with hematoma volume [20]. Namely, CSF metabolome from SAH patients with Hunt-Hess Scale ranges ≥III could be considered at identical baseline. In the present study, the effect of sedatives was separated from that of SAH severity, for we only chose patients with Hunt-Hess Scale above III and the baseline metabolome was therefore comparable.

For these reasons, we hypothesized that the sedatives differentially modulate brain metabolism in the acute phase of SAH, and therefore influence their long-term outcome. We therefore undertake this observational study to portray the CSF metabolic profile of SAH patients treated with different sedatives (midazolam, propofol and dexmedetomidine), to compare their metabolome, and to identify possible biomarkers of satisfying sedation as well as outcome of the patients.

## Methods

### Subjects and sample collection

As an observational study, we collected CSF samples from SAH patients admitted to the Neurosurgical Critical Care Unit in Nanjing Drum Tower Hospital Department of Neurosurgery between September 2017 to December 2018. The CSF was sampled within 7 days after SAH onset. The study adhered to ethical guidelines, and was approved by the Ethical Committee of Nanjing Drum Tower Hospital. Written informed consent was obtained from all individual participants or their legal guardians included in the study. Hunt-Hess scale [36], Fisher grade [37], World Federation of Neurological Societies (WFNS) scale [38] of the patients were evaluated by three neurosurgeons independently, and the most frequent scores were recorded. Richmond Agitation-Sedation Scale (RASS) [39] was implemented by trained bedside nurses. Patients whose Hunt-Hess scales were above III and agitated (RASS at + 2 to + 4) were included in the study. Patients in sedated groups underwent midazolam, propofol or dexmedetomidine light sedation, whereas those in control groups underwent no sedation when CSF was sampled. CSF was collected simultaneously when lumbar puncture or CSF drainage (through external ventricular drain or lumbar cistern drainage) was needed to alleviate hydrocephalus or drain out bloody CSF. And CSF was sampled between 24 h to 72 h after the initiation of sedation. The samples were collected from the discarded portion of CSF when lab tests were needed. Patients (a) whose CSF was not drained out or not collected successfully (b) who underwent sedation and did not meet desirable effect (RASS between − 2 and 1) or whose RASS was lower than − 2 when CSF was collected (c) who utilized more than one sedative were excluded from the study. Patients treated with sedatives were followed up at 1 year after discharge, and Glasgow Outcome Scale (GOS) was utilized to evaluate the outcome of these patients.

### Metabolomic study

The CSF samples were centrifuged at 3000 rpm and the supernatants were transferred to fresh conical tubes and stored at − 80 °C before use. The samples were applied to high performance liquid chromatography coupled with mass spectrometer as previously described [20]. Compounds were identified after annotation procedure. After internal standard calibration, peak areas were applied to statistical analysis.

### Data processing and statistical analysis

Statistical analyses were performed using MetaboAnalyst (Version 4.0, Xia Lab, McGill University, Canada) [40] and SPSS statistics software (Version 19, IBM, United States). Metabolite data of groups were compared using analysis of variance (ANOVA) and Scheffe post hoc test. Sparse partial least squares discriminant analysis (PLS-DA) was utilized when distinguishing metabolic profiles from different CSF groups from SAH patients, and peak areas were normalized by the sum and auto-scaled. The model robustness was assessed. Significantly altered metabolites were then applied to subsequent pathway analysis, metabolite set enrichment analysis and biomarker analysis. Biomarker analysis was presented through receiver operating characteristic (ROC) curve. The data were retrieved in Kyoto Encyclopedia of Genes and Genomes (KEGG) database and the Small Molecule Pathway Database (SMPDB) to identify the significantly affected metabolic pathways. In the statistical analysis, *p* value less than 0.05 was considered significant.

## Results

### Participants

From September 2017 to December 2018, we included 42 SAH patients with Hunt-Hess Scale above III (III, IV and V). Those patients utilized single or none sedative agent. Among the patients, 11 were sedated merely with midazolam, 13 with propofol and 13 with dexmedetomidine, while 5 underwent no sedation. The patient characteristics were shown in Table 1. We then compared the CSF metabolite profiles from patients treated with different sedatives. For pyruvic acid, 2-phosphoglyceric acid and 3-phosphoglyceric acid levels were indicators of severe SAH [20], we firstly compared the concentrations of the three metabolites. There was no difference among different groups (data not shown), indicating there was no significant baseline disparity in SAH severity.

**Table 1:** Patient Characteristics

	No Sedation	Midazolam	Propofol	Dexmedetomidine	*P* value
Number of Patients	5	11	13	13	
Age (years, Mean ± SD)	65.8 ± 7.4	63.3 ± 13.5	61.5 ± 14.2	62.8 ± 11.7	0.94
Gender					
Male	1	6	7	6	0.85
Female	4	5	6	7	
Hunt-Hess Scale					
III	4	6	8	9	0.76
IV	1	5	5	4	
Fisher Grade					
2	1	1	2	2	0.94
3 ~ 4	4	10	11	11	
WFNS SAH grading scale					
II	3	2	4	4	0.42
III ~ V	2	9	9	9	
Glasgow Outcome Scale					
Poor (1 ~ 4)		8	9	9	0.98
Good (5)		3	4	4	
RASS Before Sedation					
+ 2	2	3	6	7	0.92
+ 3	2	6	5	4	
+ 4	1	2	2	2	
RASS After Sedation					
+ 1		3	5	6	0.81
0		5	4	5	
-1		3	4	2	
Other Pharmacological Interventions					
Opioid Analgesics	2	2	4	4	0.76
Non-Opioid Analgesics	2	2	4	4	0.76
Mannitol	2	6	9	8	0.55
Hyperosmotic Saline	2	7	7	7	0.79
Insulin	3	9	8	10	0.31
Antihypertensive Agents	3	9	10	11	0.22
Vasoactive Agents	2	10	10	9	0.15
Comorbidity					
Acute heart failure	2	7	7	7	0.79
Secondary Hypopituitarism	3	7	6	9	0.35
Hypertension	1	2	4	3	0.90
History of stroke or TIA	0	3	4	4	0.49

**Fig. 1:**
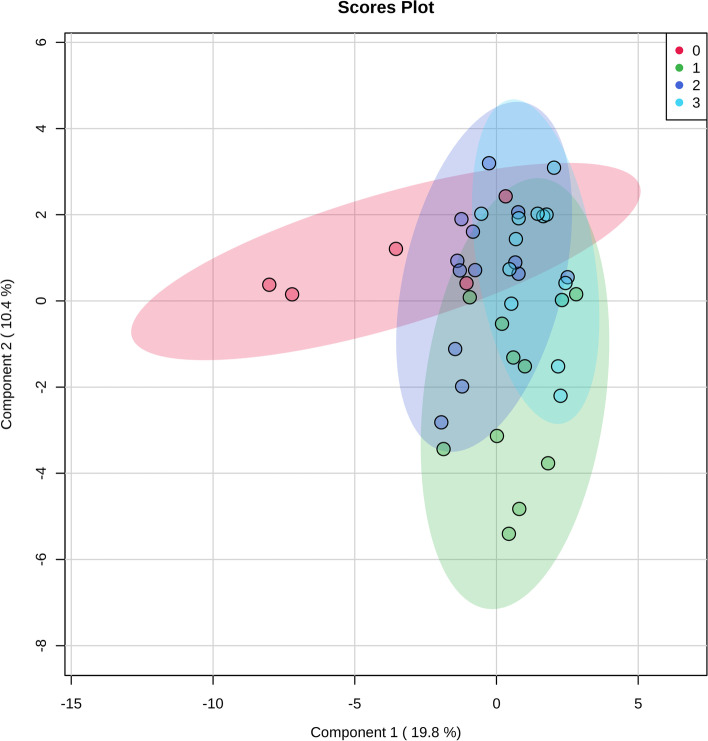
Overview of sedatives-altered CSF metabolome: 2-dimension score plot. 0, control; 1; midazolam; 2, propofol; 3, dexmedetomidine. Component 1 and 2, dimensions of the PLS-DA model

To acquire a global view of if sedatives change CSF metabolome, we compared the metabolite data of the four groups. In Fig. 1, sparse PLS-DA results showed that CSF metabolome in control group (Group 0) exerted a relatively separating trend from that of the sedatives (Group 1 ~ 3), whereas metabolomic patterns of the sedative groups were partly converged. Considering the complexity of the metabolomic data, we decided to assess metabolite profile of each sedative with that of the control group to apply to two-group analysis, to further elucidate how sedative change CSF composition, which pathways are interfered, and which biomarkers represent the alterations of CSF metabolome.

### Midazolam upregulates the level of pentose phosphate pathway metabolites in CSF of patients with SAH

**Fig. 2:**
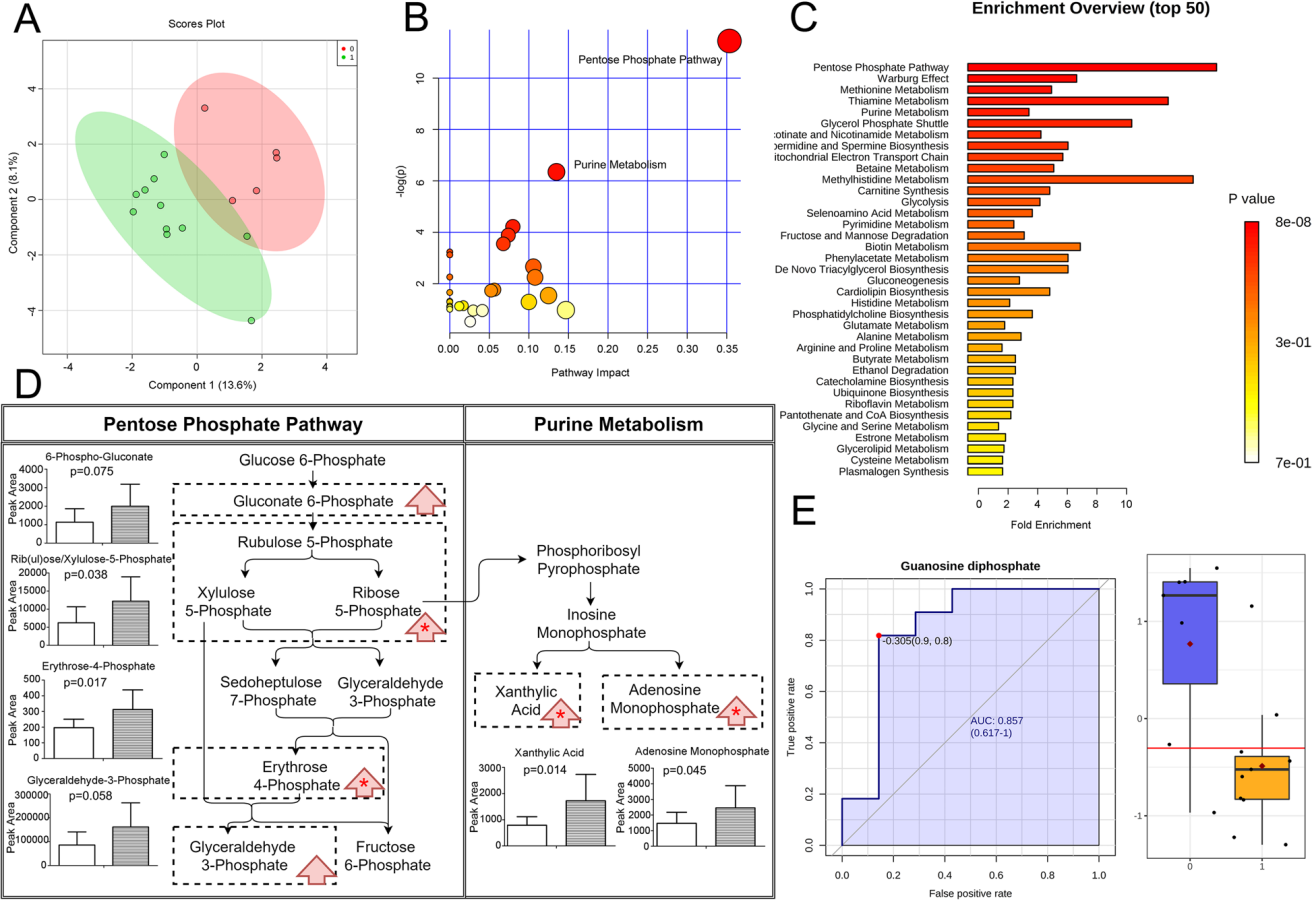
Midazolam altered CSF metabolome in SAH patients. **A** Overview of CSF metabolome in midazolam and control groups: 2-dimension score plot. 0, control; 1; midazolam; Component 1 and 2, dimensions of the PLS-DA model. Pathway analysis (**B**) and metabolite set enrichment analysis (**C**) presented the most influenced metabolic pathway by midazolam. Significantly altered metabolites were listed in (**D**), left column: control; right column: midazolam; red arrow: trend; *: statistical significance. **E** CSF biomarker of midazolam sedation, with ROC curve and cut-off value

To clarify if sedatives alter brain metabolism and CSF composition, we firstly compared CSF metabolome between SAH patients treated with midazolam or no sedation. As shown in Fig. 2A, midazolam treatment separated the CSF metabolite pattern from that of the control group. We then applied the significantly altered metabolites to pathway analysis, to identify the most affected metabolic pathway within the brain/blood and presented in the CSF. Pentose phosphate pathway was the most significantly interfered pathway, with the highest impact (*p* = 0.0000107, impact = 0.35348, Fig. 2B). Meanwhile, in Fig. 2C, metabolite set enrichment analysis, which was based on SMPDB and KEGG databases, also suggested that pentose phosphate pathway was the most biologically meaningful pathway (*p* = 0.00000787).

To further ensure whether midazolam inhibited or upregulated pentose phosphate pathway, we compared the peak intensity of metabolites identified by sparse PLS-DA and one-way ANOVA. As listed in Fig. 2D, D-rib (ul)ose/xylulose-5-phosphate and D-erythrose-4-phosphate, which are major components in pentose phosphate pathway, increased in CSF of patients treated with midazolam.

Meanwhile, pathway analysis showed that purine metabolism was also influenced by midazolam treatment, with the impact at 0.13481 (*p* = 0.00175, Fig. 2B). Apart from ribose 5-phosphate, levels of adenosine monophosphate (AMP) and xanthylic acid, which are components in purine metabolism, were significantly elevated. (Fig. 2D).

Univariate biomarker analysis demonstrated that reduced guanosine diphosphate (GDP) was the significant CSF indicators of midazolam treatment (Fig. 2E, AUC of ROC 0.857, 95%CI 0.617—1, *p* = 0.00506). Multivariate biomarker analysis did not show significant results.

The elevation of AMP and decreased GDP demonstrated that adenine nucleotide synthesis was significantly enhanced while guanine nucleotide synthesis was not comparably up-regulated. Adenine nucleotide synthesis was inhibited by the accumulation of AMP, independent of regulating guanine metabolism. Meanwhile, guanine nucleotide synthesis was not impaired and was even augmented, for decreased GDP level was the CSF indicator of midazolam treatment.

To conclude, the administration of midazolam significantly augmented metabolite levels of pentose phosphate pathway, while purine metabolism was likely to be slightly affected, as adenine nucleotides synthesis was enhanced. GDP concentration was the sensitive CSF metabolic marker of midazolam administration when sedation was desirable. These changes presented in CSF indicate the corresponding metabolic alterations in the ECSB, and at least partly, the brain.

### Propofol increases the level of pentose phosphate pathway metabolites in CSF of patients with SAH

**Fig. 3:**
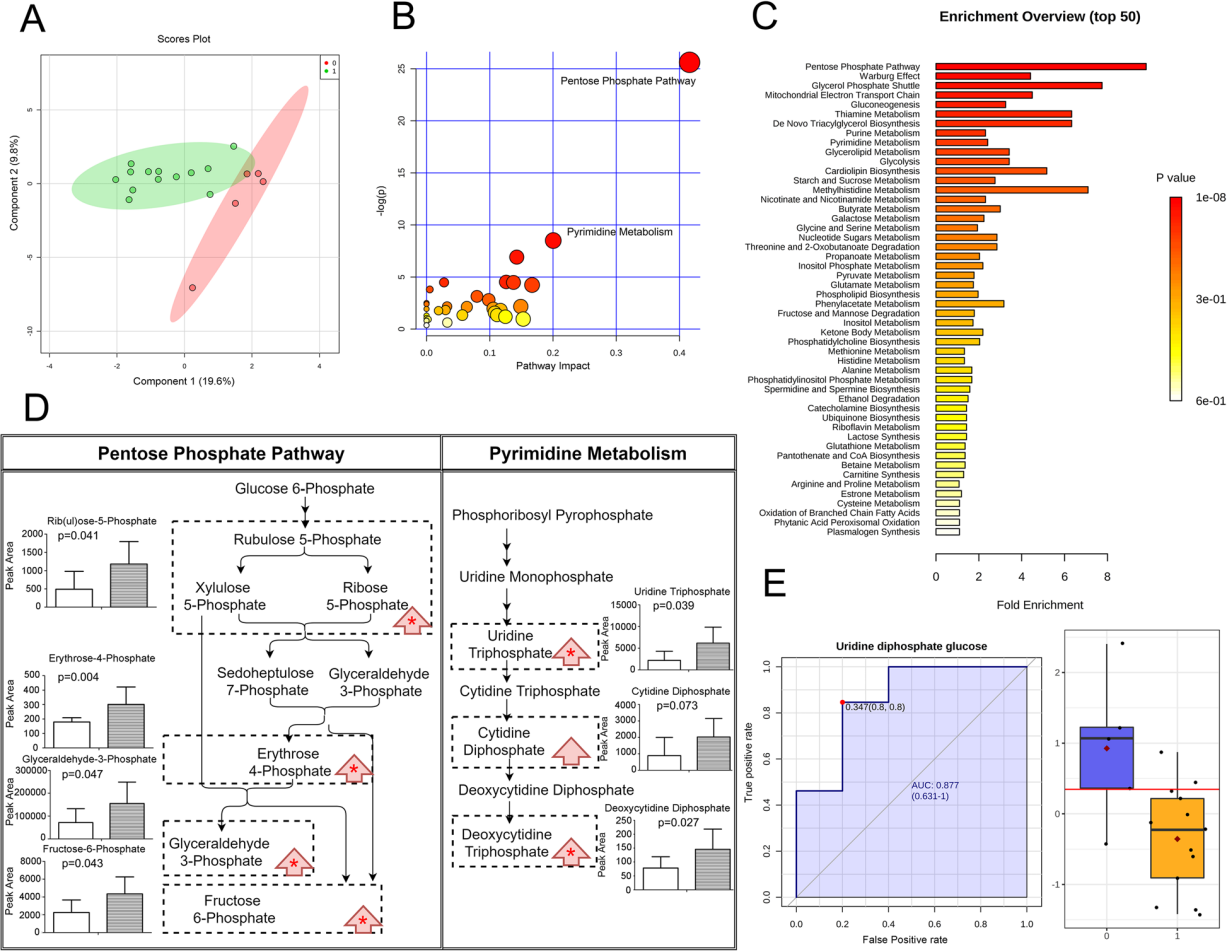
Propofol altered CSF metabolome in SAH patients. (**A**) Overview of CSF metabolome in propofol and control groups: 2-dimension score plot. 0, control; 1; propofol; Component 1 and 2, dimensions of the PLS-DA model. Pathway analysis (**B**) and metabolite set enrichment analysis (**C**) presented the most influenced metabolic pathway by propofol. Significantly altered metabolites were listed in (**D**), left column: control; right column: propofol; red arrow: trend; *: statistical significance. **E** CSF biomarker of propofol sedation, with ROC curve and cut-off value

To investigate how propofol alter brain metabolism and CSF composition, we meanwhile analyzed CSF metabolome of poor-grade SAH patients treated with propofol or no sedation. Sedating with propofol was able to change the CSF metabolome, apart from that of the control group, as shown in Fig. 3A. The significantly altered compounds identified by sparse PLS-DA and one-way ANOVA were submitted to pathway analysis, and pentose phosphate pathway was the most significantly affected (*p* = 0.00000000000746, impact = 0.41604, Fig. 3B). Metabolic set enrichment analysis also demonstrated that pentose phosphate pathway was the most affected metabolic pathway altered in CSF of SAH patients (*p* = 0.000000951, Fig. 3C).

By comparing metabolite concentrations within the CSF involved in pentose phosphate pathway, we discovered that CSF D-rib (ul)ose/xylulose-5-phosphate, D-glyceraldehyde 3-phosphate, D-erythrose 4-phosphate, fructose 6-phosphate levels significantly increased. (Fig. 3D) To conclude, pentose phosphate pathway metabolite levels were upregulated in patients treated with propofol.

Moreover, in the pathway analysis, pyrimidine metabolism was the secondly most influenced (*p* = 0.000203, impact = 0.20046, Fig. 3B). Among metabolites in pyrimidine metabolism, uridine triphosphate and deoxycytidine triphosphate levels were significantly elevated (Fig. 3D), suggesting that pyrimidine metabolism was promoted, as reflected in CSF/ECSB.

We also applied the metabolic data to biomarker analysis to search for appropriate indicators of propofol sedation. Multivariate analysis did not reach a significant outcome. Univariate analysis showed that decreased uridine diphosphate glucose (UDP-Glc) level represented satisfying propofol sedation (Fig. 3E, AUC of ROC 0.877, 95%CI 0.631—1, *p* = 0.00980).

To conclude, propofol interfered with the CSF metabolome in SAH patients by augmenting pentose phosphate pathway and pyrimidine metabolism metabolites. CSF uridine diphosphate glucose was the biomarker of the sedating effect of propofol.

### Dexmedetomidine slightly inhibits pentose phosphate pathway and strongly affects pyrimidine metabolism in CSF of patients with SAH

**Fig. 4:**
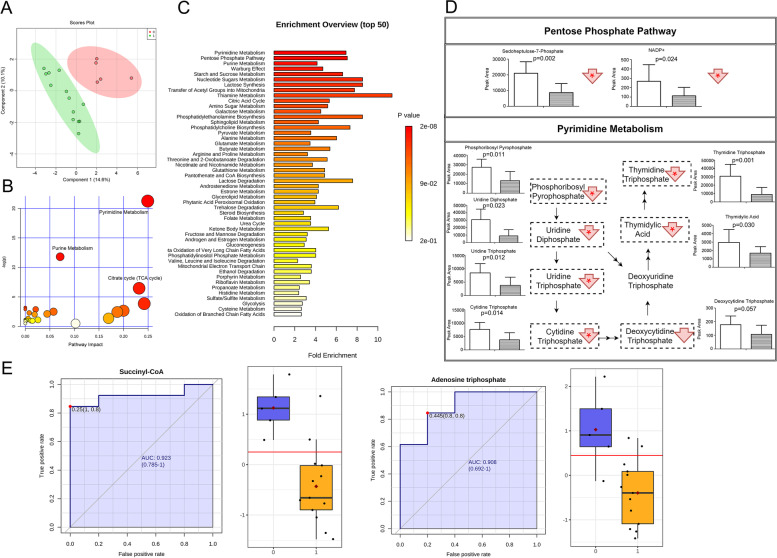
Dexmedetomidine altered CSF metabolome in SAH patients. **A** Overview of CSF metabolome in dexmedetomidine and control groups: 2-dimension score plot. 0, control; 1; dexmedetomidine; Component 1 and 2, dimensions of the PLS-DA model. Pathway analysis (**B**) and metabolite set enrichment analysis (**C**) presented the most influenced metabolic pathway by dexmedetomidine. Significantly altered metabolites were listed in (**D**), left column: control; right column: dexmedetomidine; red arrow: trend; *: statistical significance. **E** CSF biomarker of dexmedetomidine sedation, with ROC curve and cut-off value

Dexmedetomidine is one of the major sedatives commonly applying to neuro-critically ill patients. We discovered deviating metabolic pattern of CSF of dexmedetomidine-treated SAH patients from those with no sedation. (Fig. 4A).

In term of pentose phosphate pathway, pathway analysis reveals that pentose phosphate pathway metabolites in CSF were affected by dexmedetomidine treatment, however, it did not meet the statistical significance (*p* = 0.055632), and the impact was as low as 0.02108, as shown in Fig. 4B. Metabolic set enrichment analysis also demonstrated that pentose phosphate pathway (*p* = 0.000112, Fig. 4C) was the biologically meaningful and significant cytoplasmic process, which was in generally accordance with pathway analysis. Among pentose phosphate pathway metabolites, D-sedoheptulose 7-phosphate and nicotinamide adenine dinucleotide phosphate (NADP) were significantly decreased, and others were not altered (Fig. 4D).

Conversely, pyrimidine metabolism was the most significantly altered metabolic pathway (*p* = 0.000000000594), with the highest impact at 0.24922 in pathway analysis (Fig. 4B). Metabolic set enrichment analysis also demonstrated that pyrimidine metabolism was the most influenced metabolic pathway reflecting CSF metabolome alterations in SAH patients (*p* = 0.0000000168, Fig. 4C).

As shown in Fig. 4D, metabolites related to pyrimidine metabolism was decreased, during which phosphoribosyl pyrophosphate (PRPP), uridine triphosphate (UTP), uridine 5′-diphosphate, cytidine triphosphate (CTP), deoxycytidine triphosphate, 5-thymidylic acid and thymidine 5′-triphosphate were significantly down-regulated.

Citrate cycle (impact = 0.2317, *p* = 0.0015628) was also significantly interfered (Fig. 4B), for succinyl-CoA and adenosine triphosphate (ATP) levels were significantly down-regulated (refer to Fig. 4C). However, citrate cycle occurs mainly in mitochondria, and CSF might not completely reflect mitochondrial metabolite concentration alteration through CSF-ECSB substance exchange. Therefore, we only take metabolic processes located in cytosol in consideration.

In addition, purine metabolism (impact = 0.07093, *p* = 0.0000074485, Fig. 4B; *p* = 0.000138, Fig. 4C) biologically meaningful and significant cytoplasmic process, which was in generally accordance with pathway analysis; however, purine metabolism was of relatively low impact.

Moreover, as shown in Fig. 4E, univariate biomarker analysis showed that down-regulated succinyl-CoA (AUC of ROC 0.923, 95%CI 0.785—1, *p* = 0.000810) and ATP (AUC of ROC 0.908, 95%CI 0.692—1, *p* = 0.00315) concentrations were the significant CSF indicators of dexmedetomidine treatment. Multivariate biomarker analysis did not show significant results.

Therefore, dexmedetomidine affected CSF metabolome in SAH patients by inhibiting pyrimidine metabolism, and meanwhile pentose phosphate pathway was also slightly down-regulated. The suppressed energy supply, as represented by diminished succinyl-CoA and adenosine triphosphate concentrations, was the biomarker of dexmedetomidine sedation. The modulation of pentose phosphate pathway was divergent in CSF of patients treated with midazolam, propofol and dexmedetomidine.

### Succinyl-CoA level in CSF was significantly down-regulated in SAH patients with favorable outcome

One-year GOS of the sedated SAH patients was analyzed with their metabolomic data. GOS between 1 and 4 was classified as unfavorable outcome and the corresponding CSF GDP, UDP-Glc, succinyl-CoA and ATP levels were submitted to biomarker analysis. It showed an association with decreased succinyl-CoA concentration and favorable outcome (AUC of ROC: 0.708, 95% CI: 0.524–0.865, *p* = 0.029333, sensitivity 0.7, specificity 0.7) in poor-grade SAH patients. Interestingly, down-regulated CSF succinyl-CoA level was also one of the biomarkers of dexmedetomidine sedation. Dexmedetomidine, although failed to bring a better prognosis to patients in the present study (Table 1), is previously reported as a beneficial factor of SAH outcome [10].

## Discussion

The present study offered the metabolomic perspective to discover how different sedatives alter cerebrospinal fluid metabolite profile in SAH patients and the potential of these agents in affecting the outcome of the patients. Midazolam and propofol significantly up-regulated CSF metabolites of pentose phosphate pathway, whereas dexmedetomidine exerted a slight inhibitory trend. In term of nucleotide biosynthesis, as reflected in the CSF, midazolam and propofol were able to promote purine and pyrimidine metabolism respectively, whereas dexmedetomidine diminished pyrimidine nucleotide biosynthesis, down-regulating the CSF levels of its metabolites. Decreased levels of GDP, UDP-Glc and succinyl-CoA plus ATP were corresponding biomarkers of desirable midazolam, propofol and dexmedetomidine sedation, and down-regulated CSF succinyl-CoA concentration was associated with better Glasgow Outcome Scale at one-year follow-up. However, the present study failed to find a better outcome in the dexmedetomidine group (Table 1).

Through global CSF neurochemical monitoring, we firstly found that midazolam shifted the CSF metabolome away from the control group and up-regulated the CSF metabolites in the pentose phosphate pathway. Previous studies have not link pentose phosphate pathway to midazolam action; on the contrary, they mainly focus on glucose oxidation, which is the first of the research highlights, for its compelling importance in supporting normal cerebral function [35, 41]. It is proved that midazolam sedation keeps the lactate and pyruvate production intact [23]. In animal experiment, cerebral oxygen consumption was also found unchanged in midazolam-sedated rats [42]. It is verified that midazolam regulates ECSB glucose level (> 1 mmol/L) in a dose-dependent manner, and keeps critical glucose concentration (< 1 mmol/L) unaffected [35]. The limitation of the previous hypothesis and detection makes it uncapable to discover the involvement of pentose phosphate pathway. The elevated level of pentose phosphate pathway flux, suggesting that the brain was more inclined to anabolism, which was in accordance with the up-regulated flux of purine synthesis.

Interestingly, the adenylates and guanylates biosynthesis was not parallelly modulated by midazolam. In the present study, the synthesis of adenine nucleotides was enhanced, whereas that of guanine nucleotides levels were decreased. This imbalance was also verified in other situations [43]. For guanylates are utilized in the faster way than adenylates [44], guanine nucleotides depletion was supposed to be the rapid access to the sedative-induced deoxyribonucleic acid (DNA) synthesis, whereas the large adenine nucleotides pool kept stable, supplying abundant substrates to DNA synthesis. And this hypothesis could partly explain why down-regulated GDP level characterized the desirable midazolam treatment in SAH patients.

Similarly, propofol also altered CSF metabolome from that of the control group, and the metabolomic pattern was parted congregated with that of the midazolam group. Pentose phosphate pathway was the most significantly augmented metabolic pathway, and was associated with the up-regulated pyrimidine biosynthesis by providing essential substrates. The connection between propofol and pentose phosphate pathway has not been reported previously [45], and its potential of regulating nucleotide biosynthesis might be underestimated.

Furthermore, UDP-Glc, as the substrate of glycogen synthase providing the glucose molecule, suggested the tendency of weakened brain/systemic glycogen synthesis. Notably, in the brain, glycogen metabolism in the glia provides essential energy substrate to neurons [46]. The present study provided multiple clues of further exploration of the propofol pharmacology.

The action of dexmedetomidine was the opposite. Unlike midazolam or propofol, this sedative slightly but not significantly down-regulated the level of metabolites in pentose phosphate pathway. NADP is the key regulator of the oxidative phase of pentose phosphate pathway, and its level in CSF was significantly down-regulated after dexmedetomidine treatment. The pentose phosphate pathway (PPP) was weakened, and the production of ribose-5-phosphate through PPP, which supports nucleotide biosynthesis, was thereby insufficient. Importantly, nucleotide synthesis is essential in maintaining normal brain function [47].

The CSF metabolome, in dexmedetomidine-treated group, was diverged from that of the control group, and the diminished pyrimidine synthesis was the most influenced metabolic pathway. Given the pentose phosphate pathway was mildly inhibited, its assistance of pyrimidine synthesis was thereby weakened. Thus, the decreased concentration of the CSF metabolites in pyrimidine metabolism could at least partly explained. In pyrimidine metabolism, ATP and PRPP activate carbamoyl phosphate synthase (CPS) II and UTP inhibits the enzyme, and CTP inhibit CTP synthase [48]. For ATP, PRPP, CTP and UTP levels decreased in CSF of dexmedetomidine-treated patients, we could conclude that upstream targets instead of CPSII or CTP synthase were regulated. Previous studies have not connected dexmedetomidine to cerebral pentose phosphate pathway or pyrimidine synthesis, and the present discovery suggested the necessity to more detailed elucidation of dexmedetomidine action.

In addition, decreased succinyl-CoA and ATP levels are sensitive and specific biomarkers of dexmedetomidine sedation. Meanwhile, alteration in CSF succinyl-CoA concentration was also associated with patient outcome, which might be the key compound linking dexmedetomidine pharmacology and attenuation of neurological deficit in poor-grade SAH patients. Moreover, as crucial indicators of energy supply, the two reduced biomarkers suggested a relatively low activity of citrate cycle. However, the alteration of this mitochondrial process, which could not be completely presented in the ECSB/CSF, should be carefully studied in the future.

There were limitations of this study. The mechanisms underlying the observations in the present study require more research, focusing on how sedatives alter metabolic pattern of the brain on molecular level and whether drug action on neuron and glia differs. Secondly, the metabolomic approach has its own defect that it is not able to tell regional or local data [19]. For instance, pentose phosphate pathway activity is divergent in different brain regions [49], indicating the importance of potential role of regional metabolic disparity. The overall metabolomic study might omit the variance. Meanwhile, for the sample size was limited, the study was not able to tell a dose-dependent alteration in CSF metabolic profile. In addition, for CSF acquisition before as well as after sedation might not be feasible for the same patients in the present study, pair-matched design was not applied. However, metabolic profiling of brain metabolism before the initiation of sedation and after in the same patients should be considered in further investigation, to maximumly reduce the interindividual variation.

## Conclusion

By analyzing CSF metabolome of poor-grade SAH patients sedated with midazolam, propofol and dexmedetomidine, we discovered that these drugs changed CSF metabolic profiles in divergent ways. The influence of CSF composition by SAH severity was excluded by baseline calibration. Midazolam and propofol up-regulated pentose phosphate pathway metabolites and enhanced purine and pyrimidine synthesis respectively. On the contrary, dexmedetomidine down-regulated pentose phosphate pathway metabolites and attenuated pyrimidine synthesis. We also linked satisfying sedation with their CSF metabolites as biomarkers. Down-regulated GDP and UDP-Glc levels were biological indicators of desirable midazolam and propofol sedation, whereas diminished succinyl-CoA and ATP concentrations represented that of dexmedetomidine treatment. Succinyl-CoA level down-regulation was also associated with better clinical outcome of these poor-grade sedated SAH patients. How sedatives alter brain metabolism and how these metabolic profile alterations associate with the prognosis of the SAH patients remained to be further elucidated.

## Data Availability

The datasets used and/or analyzed during the current study are available from the corresponding author on reasonable request.

## Abbreviations

SAH: Subarachnoid hemorrhage
CSF: Cerebrospinal fluid;
PET: Positron Emission Computed Tomography
^1^HMR: Proton magnetic resonance
ECSB: Extracellular space of the brain
RASS: Richmond Agitation-Sedation Scale
GOS: Glasgow outcome scale
PLS-DA: Partial least squares discriminant analysis
ROC: Receiver operating characteristic
KEGG: Kyoto Encyclopedia of Genes and Genomes
SMPDB: Small Molecule Pathway Database
AMP: Adenosine monophosphate
GDP: Guanosine diphosphate
UDP-Glc: uridine diphosphate glucose
AUC: Area under the curve
NADP: Nicotinamide adenine dinucleotide phosphate
PRPP: Phosphoribosyl pyrophosphate
UTP: Uridine triphosphate
CTP: Cytidine triphosphate
ATP: Adenosine triphosphate
CPS: Carbamoyl phosphate synthase
